# Examining Associations between Health Information Seeking Behavior and Adult Education Status in the U.S.: An Analysis of the 2012 PIAAC Data

**DOI:** 10.1371/journal.pone.0148751

**Published:** 2016-02-16

**Authors:** Iris Feinberg, Jan Frijters, Vicki Johnson-Lawrence, Daphne Greenberg, Elena Nightingale, Chelsea Moodie

**Affiliations:** 1 Adult Literacy Research Center at College of Education and Human Development, Georgia State University, Atlanta, Georgia, United States of America; 2 Child and Youth Studies, Brock University, Toronto, Canada; 3 Public Health & Health Sciences, University of Michigan-Flint, Flint, Michigan, United States of America; 4 Educational Psychology, Special Education, and Communication Disorders, Georgia State University, Atlanta, Georgia, United States of America; 5 Center for the Study of Adult Literacy, Georgia State University, Atlanta, Georgia, United States of America; Leibniz Institute for Prevention Research and Epidemiology (BIPS), GERMANY

## Abstract

This paper presents data from the Program for the International Assessment of Adult Competencies with a focus on the interrelationships among health information seeking behavior (HISB), and health status or use of preventive health measures for U.S. adults both with and without a high school diploma. Key results of ordinal and binary logistic regression analyses indicated that, after controlling for demographic factors, (1) adults with a high school diploma use more text-based health information sources while adults without a high school diploma use more oral sources, (2) using the Internet as a source of health information is more strongly related to reporting excellent/very good health status than having a high school diploma, (3) those without a high school diploma who use the Internet report the largest increase in health status over any other health information source, and (4) for those with learning disability or vision problem, a high facility in reading English is an important predictor of whether the Internet is used as a health information source. The Internet appears to play a key role in both enhancing health status and enabling use of preventive measures for those with and without a high school diploma; although, individuals without a high school diploma who use the Internet for health information derive substantial benefit in health status.

## Introduction

A key component of high-quality healthcare is patient-centered care (PCC) in which patients and their providers work together to make decisions about health care and disease management [1]. In order to participate in their care, patients must have adequate health literacy, which includes the ability to read, listen, ask questions, and draw conclusions from health-related information [2,3]. Health Literacy is a concept that is complex and dynamic, and takes into account how people access, understand and use health information and health care in everyday life and in medical situations. There are a number of individual factors (age, socioeconomic status, race) and societal factors (health disparities, community norms, culture) that can affect health literacy, however, adults with low literacy and low education status are less likely to have the basic literacy skills that correspond to adequate health literacy, and therefore are more likely to have low PCC and poor health outcomes in both primary prevention and chronic disease [3–6].

Adults at many education levels struggle to understand medical statistics, medication dosage requirements, and basic health concepts such as daily nutritional values [4,6,7]. Education levels are often used as a proxy for literacy skills, however, over 80% of adults with low-level literacy, numeracy, and problem solving skills do have a high school diploma (OECD). We also know that seeking health information is not only related to literacy and education levels, but also has many other predisposing characteristics including gender, age, education level, general and health literacy levels, pre-existing health conditions, and race [7,8,9]. However, people who do have a high school diploma, regardless of their literacy levels and other socio-demographic factors, are more likely to seek and use health information. Education levels and literacy levels are both strongly linked to health outcomes [10,11].

Health information seeking behavior (HISB) can be enacted through print, visual, or oral media; health information can also be accrued either actively or passively. Although there are many studies that consider HISB for specific diseases and health conditions, very few have addressed the role and influence of HISB in a population with diverse characteristics who may have no specific diagnosis or disease [3,6, 9]. We are interested in knowing from which sources those with and without a high school diploma seek health information, and if there is any further association with health status and use of preventive measures. The purpose of this study is to gain an understanding about HISB and its determinants with adults of different education levels in the United States through analysis of the Program for International Assessment of Adult Competencies (PIAAC) data. The PIAAC is an international household survey conducted under the auspices of the Organization for Economic Cooperation and Development (OECD), in 26 countries. Each country was allowed to add five minutes of questions; the United States added questions relating to health information seeking and health behaviors as part of the country data. Our focus is therefore on the US data since no other country in the PIAAC study asked questions about HISB. The data provide us with a unique opportunity to understand how adults seek health information while controlling for demographic and socioeconomic factors [10]. We address this question by asking the following research questions:

1. Is there a difference between HISB for individuals with and without a high school diploma?
2. Is there an association between HISB, health status or use of preventive measures for individuals with and without a high school diploma?

### Education and Health

Educational attainment matters for health—past studies have shown that education levels are linked with health through health knowledge and behaviors, literacy levels, employment status, insurance status, and a variety of other social and psychological factors [11–15]. People with more education report having lower morbidity from common acute and chronic diseases; likewise, they are also more likely to exercise and obtain preventive care [16–18]. Those with higher educational attainment also tend to have higher health literacy levels, which enables them to better access, understand, and communicate actionable health information [8, 13, 17, 19–21]. While acknowledging the role that genetic traits, demographic factors, and socioeconomic status have on health outcomes, we frame our study within the persistent association between educational attainment and health because of the influence of education level on accessing and understanding health information [11,13,17,22,23].

One well-described and important factor that links education and health outcomes is economic status [11,17,23–30]. Those with higher education are more likely to have higher income, greater wealth, more stable employment, better health insurance, and access to health care [11,18, 22, 25,31–32]. While it is not clear if the pathway from education to health is causal, a consistent finding within economic, education, and health research is that those with higher incomes tend to have better self-reported health, report fewer physical and mental limitations, and are more likely to live in communities that support healthy lifestyles [33–35]. Those with higher education also tend to have well-developed cognitive skills (e.g., reading, writing, and reasoning), which allows them to accrue greater knowledge about health matters and use that information to engage in effective risk assessment and medical decision making [36,37]. Access to health resources provides more information about preventive health behaviors and the benefits of a healthy lifestyle. Individuals with greater access to these sources due to higher economic and education status are more likely to engage in preventive health measures and live a healthy lifestyle [33–35,38,39].

Another key factor that links education and health outcomes is the development of non-cognitive skills that may promote better health outcomes through active decision making about appropriate health behaviors [40,41]. For example, adults with lower educational attainment are less likely to have a developed sense of personal control, which is highly related to better health through development of traits such as delayed gratification and persistence [42–44]. Personal control is a key element in self-efficacy, which affects health directly through psychological factors (e.g., beliefs, coping, help seeking) and indirectly through actual behaviors (e.g., “if I don’t believe I can quit smoking, I will have another cigarette”) [45–47]. For example, a key psychological prerequisite to seeking out healthy behaviors or preventive screening is the belief that good health outcomes are within one’s personal control. Health behaviors play a strong role in explaining health and illness, particularly as they relate to illness onset, help-seeking, illness management, and health outcomes [45,47].

Literacy is a person’s ability to read, write and speak and is often measured in health care settings by general literacy tests such as the Wide Range Achievement Test (WRAT) or by health literacy tests such as the Rapid Estimate of Adult Literacy in Medicine (REALM) or the Test Of Functional Health Literacy in Adults (TOFHLA) [3,5,6,14, 35,48–50]. People with less education have a high prevalence of literacy difficulties whether measured by general or health literacy tests. There is a strong association between low reading skills and health outcomes which is thought to be primarily due to a general lack of knowledge about health and a lack of understanding about health services [35,40,51,52]. In addition, health literacy studies indicate that grade level equivalents in reading in a health context may be considerably lower than grade level equivalents in general reading [35,48,52–55].

The abovementioned tests directly assess reading skills only and do not take into consideration other literacy assets such as memory, sight-reading, or problem solving. These more general skills may help people navigate the complex world of health and healthcare despite having low reading skills or not having a high school diploma. One general finding from the PIAAC study is that people with low literacy skills are four times more likely than those with above average literacy skills to have poor health [10].

### Education, Literacy and Health Information Seeking

Education levels also affect how one seeks and uses information. Information seeking skills are often learned and used when meeting certain objectives, like gathering information for a research project in school [56,57]. Students learn how and where to source information in the school setting, using tools such as textbooks, resource materials, Internet searching, and other information sources [56,57]. Through information acquisition, people accrue both content knowledge for and practice of problem-solving and critical thinking. Adults who have not finished high school may not have basic information seeking competencies, and may therefore not be able to know when information is needed, how to identify or locate that information, and how to use that information to problem solve [53,56,58–60]. Additionally, and depending on more specific literacy competencies, they may not have the necessary cognitive or literacy skills to be able to read and comprehend the information they do find [56–58,60].

HISB has the ability to shape health outcomes by providing access to important information for understanding and coping with a health risk, increasing involvement in medical decision making, and promoting preventive behavior and healthy behavior change [2,7–9,48,61–63]. Adults who have higher levels of education are more likely to seek information which enhances a sense of personal control through mastering content and developing stronger analytic and communication skills [53,60,63]. Knowing how adults with differing education levels engage in HISB is important because those who actively seek health information from a variety of sources are likely to use that information and be more cognitively and psycho-socially prepared to engage in medical decision-making and with the medical system [64,65].

Seeking information is also a prerequisite to using information. Those with lower education levels are less likely to have the skills or knowledge to seek health information [53,56,58,59,66]. Adults with less than a high school diploma are less likely to be knowledgeable about both preventive measures and management of sick behaviors because they are less likely to seek health information [67]. The high literacy, numeracy, and computer skill demands of health-related websites create problems for those who have low educational attainment [68]. Challenges also exist in when seeking information from health professionals—the complexity of medical language, discordance between language and literacy skills of patients and providers, and intercultural communication issues contribute to the difficulty that adults with low education levels have in participating fully in their health care [68,69].

It is important to control for a variety of socio-economic and demographic factors when analyzing the relationship between literacy and health. People with low literacy are more likely to have low income, low levels of education, and limited English proficiency [1,12,15–19, 24]. In addition, they are more likely to be Black, Native American, or Latino and more likely to be elderly [20, 23]. Those with lower levels of education may have more challenges seeking health information from written sources, and may instead choose oral sources of information across these factors [70,71]. Insurance status can also confound health information seeking: those with insurance are more likely to seek health care for non-emergent and chronic conditions which puts them in contact with health professionals more than those who do not have health insurance [72–74]. Often, first-generation immigrants struggle when seeking health information due to language barriers and lack of cultural familiarity with the US medical and health systems [75,76].

## Methods

### Study Population

Data for this study were acquired from the 2012 PIAAC dataset using the United States country-specific background questionnaire administered to a representative sample of 5,010 adults between the ages of 16 and 65. The PIAAC is an international survey conducted under the auspices of the Organization for Economic Cooperation and Development (OECD). Background questionnaires were delivered in English or Spanish; the direct assessment measures of literacy, numeracy, and problem solving in technology-rich environments were delivered in English only. Participants were only included only if there was no missing data in any of the dependent and independent variables under study in order to avoid separation of the data. Each country was allowed to add five minutes of questions to their background questionnaire. The United States included questions relating to health status, health information seeking behaviors, and use of preventive health measures.

### Eligibility

Our sample included all PIAAC participants who reported their high school diploma status, excluding the small proportion of individuals who did not report their status (2.6%; n = 125). Within our sample, 4256 had a high school diploma and 629 did not have a high school diploma.

### Variables

#### Sources of Health Information

Health information seeking behavior was established through the sources of health information utilized by the sample participants. There were eight different source variables: newspapers, magazines, Internet, radio, television, books or brochures, family members/friends/co-workers, and health professionals. For each source, participants were asked “How much information about health issues do you get from…”. The responses, “A lot”, “Some”, “A Little”, and “None” were coded on a Likert Scale from 1–4. Individuals rated each of the eight sources according to the response scale. We considered creating composite variables for related sources (e.g., Radio/TV); however, correlations among sources ranged from small to moderate, with the average gamma coefficient being .31 (γ’s ranged from .12 to .64). Based on this analysis, each source of health information was analyzed separately. HISB was the dependent variable for Research Question One and the independent variable for Research Question Two.

#### Self-Reported Health Variables

Health status was self-reported as “Excellent”, “Very Good”, “Good”, “Fair” and “Poor”. Average health status score was calculated for descriptive purposes, ranging from 1 (poor health) to 5 (excellent health). For regression analyses, health status was dichotomized as excellent/very good health compared to good/fair/poor health. Respondents answered a series of eight questions (yes/no) with regard to their use of preventive measures: “In the past year have you had a…” flu shot, mammogram, pap smear, screen for colon cancer, dental visit, vision check, screen for prostate cancer, and screen for osteoporosis. We created composite indices of preventive measure use for practices that were highly correlated. Among women, mammogram, colonoscopy, and osteoporosis screening were combined (γ = .67 and γ = .82, respectively); among men, colonoscopy and prostate screening were combined (γ = .93). There were no other strongly correlated preventive health measures.

#### Demographics

Gender, age, race and high school diploma status, first generation immigrant status and having medical insurance were determined using PIAAC variables. With regard to age, we used age groups of 24 and under, 25–34, 35–44, 45–54, and 55–65. Race was categorized into four variables: White, Black, Hispanic, and Asian/Other. For Educational Attainment, we created a variable to indicate whether or not a person had a high school diploma based on self-reported data. Immigrant status and medical insurance status variables were determined based on self-reported data.

#### Statistical Analyses

The analyses were performed using SAS v. 9.3 (Cary, NC) after downloading the PIAAC U.S. Public Use File Number 2014045 from the National Center for Education Statistics and creating the abovementioned variables (SAS, 2002–2004; U.S Department of Education, 2013). All appropriate weighting macros derived by PIAAC were utilized in order to provide population-level results adjusted for the sampling methods used in the study. By using random selection methods at each stage of sampling, this four-stage stratified area probability sample provided reliable statistics for the US population from the sampled data [10].

According to NCES Statistical Standards and IES Data Security’s rules, sample frequencies were rounded up to the nearest 10s. Descriptive characteristics of the sample were examined using frequencies and percentages for categorical measures. Associations between categorical and/or ordinal measures were assessed using chi-square tests and gamma coefficients, respectively. T-tests and Mann Whitney U tests, depending on level of measurement, were performed to compare high school diploma status on the dependent measures.

Ordinal logistic regression models were employed to examine the associations between high school diploma status and health information source outcomes. Binary logistic regression models were used to study the relationship between high school diploma status and HISB with the health and preventive measure outcomes.

A series of four models were performed that included (1) high school diploma status and the primary predictor of interest: (2) interaction terms of high school diploma status with the primary predictor; (3) we added demographic confounders to the model; and (4) we performed models stratified by high school diploma status to assess the magnitude of association between the primary predictors and the outcomes of interest when significant interactions were indicated.

## Results

Before specific research questions are addressed, we looked at general characteristics of our sample which are shown in Table 1.

**Table 1:** Frequencies for total sample, Rounded to nearest 10s per NCES Statistical Standards and IES Data Security’s rules, 2012 PIAAC Data (N = 5010).

	Actual N	Weighted %	SE
**Gender**			
Male	2260	49%	0.22
Female	2630	51%	0.22
**Age**			
Under 24	810	18%	0.38
25–34	1020	20%	0.38
35–44	950	20%	0.30
45–54	1050	22%	0.37
55+	1040	19%	0.24
**Race**			
White	3310	65%	0.91
Black	640	13%	0.11
Hispanic	560	14%	0.41
Other	370	8%	0.10
**High School Diploma**			
Yes	4256	85%	.28
No	629	15%	.28
**1st generation Immigrant**			
Yes	600	15%	0.55
No	4050	85%	0.43
**Medical Insurance**			
Yes	3860	79%	0.89
No	1010	21%	0.89
**Health Status**			
Poor	1150	24%	0.85
Fair	1620	33%	0.85
Good	1370	28%	0.87
Very Good	570	11%	0.59
Excellent	190	4%	0.25

### Research Question 1

Research question 1 asked “Is there a difference between HISB for individuals with and without a high school diploma?”

Health information sources were the dependent variable. Table 2 shows the use of health information source by education status. Usage of text-based sources (e.g., newspaper, magazine, Internet, books) was associated with having or not having a HSD. At high levels of usage, more people with a high school diploma used such sources; whereas, people without a HSD were more likely to report low usage. Compared to people with a high school diploma, a greater proportion of people without a high school diploma used oral information source (e.g., radio, television). The use of health professionals for health information was almost the same for those with (45.6%) and without (44.8%) a high school diploma. Chi-square tests showed that the distributions of each of the health information source usage significantly across high school diploma status (*p* < .05). The differences in usage represented small effects (Cramer’s V .04 to .15), with medium effect sizes for both Internet and Book usage. Given the multiple tests across the seven sources of information, the Benjamini/Hochberg [74] procedure was used to control the false discovery rate.

**Table 2:** Use of health information source by high school diploma (HSD) status, 2012 PIAAC data (N = 4885).

	A Lot	Some	A Little	None		
	Weighted %	Weighted %	Weighted %	Weighted %	Cramer’s V	χ^2^
Newspaper					.04	26.65*
HSD	7.2	27.3	31.6	33.9		
No HSD	6.2	20.2	27.6	46.1		
Magazine					.08	99.1*
HSD	9.6	36.6	32.3	21.4		
No HSD	8.4	23.5	25.0	43.0		
Internet					.15	347.8*
HSD	50.5	29.0	10.2	10.3		
No HSD	35.2	18.3	12.0	34.5		
Radio					.05	31.86*
HSD	8.4	26.9	32.3	32.5		
No HSD	11.8	22.8	23.9	41.5		
Television					.05	38.45*
HSD	27.4	37.9	23.1	11.6		
No HSD	38.4	33.8	16.2	11.6		
Books					.10	153.53*
HSD	15.8	36.5	29.3	18.4		
No HSD	11.3	21.8	26.0	41.0		
Health Professionals					.06	56.33*
HSD	45.5	32.9	15.4	6.3		
No HSD	44.8	24.2	16.6	14.4		

*Significance at *p <* .0001, controlling false discovery rate

Results of the ordinal logistic regression models are shown in Table 3. Model 1 shows the association between having a high school diploma and utilization of each of the seven health information sources, controlling for other sources of health information. People with a high school diploma were more likely to report using magazines (OR 1.37, 95% CI 1.06–1.67), the Internet (OR 2.18, 95% CI 1.86–2.55), and books (OR 1.64, 95% CI 1.33–2.03) for health information, but less likely to use television for health information (OR = 0.57, 95% CI 0.47–0.69) compared to people without a high school diploma. The magnitude of the associations between having a high school diploma and magazine, Internet, and book usage increased after additionally controlling for age, race/ethnicity, gender, immigrant status, and having medical insurance; the association between having a high school diploma and using television as a health information source was weakened (OR = 0.67, 95% CI 0.54–0.84), but remained statistically significant.

**Table 3:** Prediction of health information source use via high school diploma (HSD) status, Model 1 controlling for sources, Model 2 controlling for sources and demographics, 2012 PIAAC data (N = 4885).

Outcome Variable	Model 1: HSD + Other Sources		Model 2: + Demographics	
	OR	95% CI	OR	95% CI
Newspaper	1.10	0.89–1.36	1.11	0.89–1.38
Magazines	1.37	1.06–1.67*	1.38	1.07–1.77*
Internet	2.18	1.86–2.55*	2.97	2.46–3.59*
Radio	1.03	0.86–1.24	0.93	0.76–1.15
TV	0.57	0.47–0.69*	0.67	0.54–0.84*
Books	1.64	1.33–2.03*	1.75	1.41–2.17*
Health Professionals	0.95	0.74–1.21	0.84	0.64–1.10

* *p* < .05

We further examined significant interactions between high school diploma status and each of the health information sources, controlling for demographic measures to determine use of multiple sources. We performed analyses stratified by high school diploma status to assess the magnitudes of the interactions, and the results are shown in Table 4 (details supported by data in Table A in S1 File, Table B in S1 File, Table C in S1 File, Table D in S1 File).

**Table 4:** Directional significant associations at *p* < .05 for use of multiple health information sources for High School Diploma (HSD) status, 2012 PIAAC data (N = 4885).

	Magazines		Internet		Television		Books	
	HSD	No HSD	HSD	No HSD	HSD	No HSD	HSD	No HSD
Newspaper	POS	POS	NEG	*NS*	POS	*NS*	POS	POS
Magazines	----	----	POS	POS	POS	*NS*	POS	POS
Internet	POS	POS	----	----	NEG	*NS*	POS	*NS*
Radio	POS	*NS*	POS	*NS*	POS	POS	POS	*NS*
Television	POS	POS	*NS*	*NS*	----	----	NEG	*NS*
Books	POS	POS	POS	POS	POS	*NS*	----	----
Health Professionals	*NS*	*NS*	POS	*NS*	*NS*	*NS*	POS	POS

POS = positive association

NEG = negative association

NS = Not Significant

---- Referent

These results indicated a significant interaction of high school diploma status with health information from health professionals in relation to using magazines *(p =* .039); results of the stratified models by HSD showed no significant associations between seeking health information from a health professional and seeking health information from magazines among those with and without high school diplomas. For those without a high school diploma, there was a significant association between seeking health information from the radio and from magazines (Table A in S1 File).

Interactions of HSD with using newspapers (*p* = .05) and television (*p* = .01) as sources were detected in relation to Internet usage. Models stratified by HSD (Table B in S1 File) showed no associations between newspaper usage and Internet usage for those without a high school diploma; in contrast, among those with a high school diploma, people who used newspapers for health information a little (OR = 0.84, 95% CI 0.73–0.98) or some of the time (OR = 0.81, 95% CI 0.66–0.99) were less likely to use the Internet for health information. The associations between television usage and Internet usage were non-significant in the models stratified by HSD (Table B in S1 File).

Results of the HSD stratified models showed that more television usage was associated with greater odds of radio usage for health information, but of greater magnitude among those without (OR 4.94, 95% CI 2.03–12.03) compared to those with a high school diploma (OR 2.38, 95% CI 1.88–3.02). An interaction between HSD and Internet usage (*p* = .03) was identified in relation to television usage; individuals with a high school diploma who reported a little (OR = 0.68, 95% CI 0.52–0.90) or some (OR = 0.64, 95% CI 0.48–0.86) were more likely to report television usage for health information, compared to those who did not use the Internet. No significant association between Internet usage and television usage was found among people without a high school diploma (Table C in S1 File).

An interaction between HSD and health professional usage (*p* = .04) was also found in association with book usage (Table D in S1 File). Among respondents without a high school diploma, those who seek health information from health professionals had 3.35 times the odds of using books for health information (95% CI 1.64–6.81) compared to those who did not seek information from health professionals. Among respondents with a high school diploma, those who seek health information from health professionals had 4.87 times the odds of using books for health information (95% CI 3.55–6.68) compared to those who did not seek information from health professionals (Table D in S1 File). No other interactions between HSD with health information sources were identified.

### Research Question 2

Research Question 2 asked “Does HISB play a role in health status or in seeking preventive measures for individuals with and without a high school diploma?”

Health status was the dependent variable. We examined the average self-reported health scores by high school diploma status for each health information source, specifically comparing those who reported using the sources “a lot” to the people who reported not using the source at all (Table 5). The findings showed that those who reported using the sources “a lot” to the people who reported not using the source at all (Table 5). The findings showed average health status scores were greater across HSD levels for individuals who frequently used magazines and the Internet for health information, with a medium effect size for the Internet. With regard to the Internet, there is a greater spread in mean health status (0.89) between using and not using the Internet for those without a HSD than for those with a HSD (0.69).

**Table 5:** Average self-reported health scores by HSD status for each health information source comparing use of “A Lot” to “None”, 2012 PIAAC data (N = 4885).

	Uses Source A Lot		Does Not Use Source				
	Mean	SD	Mean	SD	*d*	*t*	*p*
Newspaper							
HSD	3.60	0.09	3.63	0.04	.01	0.4	.71
No HSD	3.16	0.15	3.16	0.08	.00	0.0	.99
Magazine							
HSD	3.69	0.06	2.52	0.05	.08	-2.7	.01*
No HSD	3.49	0.14	3.10	0.07	.06	-2.2	.03
Internet							
HSD	3.76	0.03	3.07	0.06	.31	-10.9	< .01*
No HSD	3.68	0.08	2.79	0.09	.23	-8.2	< .01*
Radio							
HSD	3.71	0.06	3.67	0.03	.02	-0.7	.51
No HSD	3.45	0.14	3.18	0.08	.05	-1.8	.08
Television							
HSD	3.54	0.04	3.71	0.06	.07	2.4	.02
No HSD	3.36	0.07	3.15	0.15	.04	-1.3	.19
Books							
HSD	3.64	0.05	3.61	0.04	.02	-0.6	.50
No HSD	3.52	0.14	3.14	0.07	.07	-2.4	.02
Health Professionals							
HSD	3.57	0.03	3.65	0.08	.03	1.1	.30
No HSD	3.32	0.07	3.21	0.13	.02	-0.7	.47

*Significance at *p* < .007, controlling false-discovery rate

We performed binary logistic regression models to examine the associations between having a high school diploma/health status/preventive measure in relation to each health information source for the entire sample as shown in Tables 6 and 7.

**Table 6:** Logistic regression models predicting health status and use of preventive measures by high school diploma status and health information source (referent category “A Lot”) for total sample, 2012 PIAAC data (N = 4885), Health Status, Flu Shot, Pap Test, Colonoscopy, Vision Exam.

	Excellent/Very Good Health Status	Flu Shot	Mammogram	Pap Test	Colonoscopy	Vision Exam
	OR (95%CI)	OR (95%CI)	OR (95%CI)	OR (95%CI)	OR (95%CI)	OR (95%CI)
HIGH SCHOOL DIPLOMA	1.3 (1.1–1.7)*	.8 (.8–1.1)	1.2 (.8–1.8)	1.57 (1.1–2.2)*	.9 (.6–1.5)	1.0 (.8–1.2)
BOOKS						
Some	.93 (.8–1.1)	.9 (.7–1.0)	1.0 (.7–1.4)	1.1 (.9–1.5)	.8 (.6–1.2)	.9 (.7–1.1)
A Little	1.1 (.9–1.2)	.1.0 (.8–1.2)	1.0 (.7–1.5)	1.4 (1.1–2.0)*	.8 (.4–1.2)	.8 (.6–1.0)
Not at All	1.0 (.8–1.3)	.8 (.6–1.0)	1.4 (.9–2.2)	1.5 (1.0–2.2)*	1.0 (.6–1.6)	.8 (.6–1.1)
HEALTH PROFESSIONALS						
Some	1.3 (1.1–1.5)	.7 (.6-.9)*	.8 (.6–1.1)	.9 (.7–1.1)	.7 (.5-.9)*	.9 (.7–1.0)
A Little	1.4 (1.1–1.6)	.4 (.3-.5)*	.6 (.4-.8)*	.6 (.5-.8)*	.5 (.4-.7)*	.6 (.5-.7)*
Not at All	1.3 (1.0–1.7)	.2 (.1-.3)*	.2 (.1-.4)*	.3 (.2-.4)*	.2 (.1-.3)*	.4 (.3-.5)*
INTERNET						
Some	.9 (.8–1.0)	1.0 (.8–1.2)	1.1 (.8–1.4)	.8 (.7–1.0)	1.0 (.8–1.2)	.8 (.7–1.0)
A Little	.7 (.6-.9)*	1.1 (.8–1.0)	1.2 (.8–1.8)	1.1 (.8–1.6)	1.2 (.8–1.7)	.8 (.6–1.0)
Not at All	.3 (.3-.4)*	1.1 (.9–1.4)	.8 (.6–1.1)	.7 (.6–1.0)	1.0 (.8–1.4)	.7 (.5-.9)*
MAGAZINES						
Some	1.0 (.8–1.2)	1.1 (.9–1.4)	1.1 (.7–1.6)	.6 (.5-.9)*	1.0 (.6–1.6)	1.0 (.7–1.2)
A Little	.9 (.7–1.2)	1.2 (1.0–1.5)*	1.0 (.7–1.5)	.6 (.4-.9)*	.9 (.6–1.5)	.9 (.7–1.2)
Not at All	.7 (.6–1.0)	.9 (.7–1.2)	.8 (.5–1.3)	.5 (.3-.7)*	.8 (.4–1.4)	.8 (.6–1.1)
NEWSPAPER						
Some	1.2 (.9–1.7)	.8 (.6–1.1)	.8 (.5–12.)	.8 (.6–1.3)	.8 (.6–1.1)	.9 (.7–1.2)
A Little	1.3 (1.0–1.7)	.6 (.5-.9)*	.7 (.5–1.2)	.9 (.6–1.4)	.9 (.6–1.4)	.8 (.6–1.1)
Not at All	1.1 (.8–1.5)	.6 (.4-.8)*	.7 (.5–1.2)	.9 (.6–1.4)	.8 (.5–1.3)	.8 (.6–1.0)
RADIO						
Some	.8 (.6–1.0)	1.1 (.8–1.5)	1.8 (1.1–3.1)*	1.0 (.7–1.4)	.8 (.5–1.4)	1.2 (.9–1.7))
A Little	.8 (.6–1.0)	1.3 (1.0–1.8)*	1.6 (1.0–2.7)*	.9 (.6–1.2)	.9 (.6–1.6)	1.2 (.9–1.7)
Not at All	.9 (.7–1.1)	1.4 (1.1–1.9)*	1.8 (1.1–2.7)*	.8 (.6–1.1)	.7 (.4–1.1)	1.3 (.9–1.7)
TELEVISION						
Some	1.3 (1.1–1.5)*	1.0 (.8–1.3)	1.1 (.8–1.7)	1.0 (.8–1.3)	.8 (.6–1.2)	1.1 (.9–1.2)
A Little	1.5 (1.3–1.8)*	1.0 (.8–1.3)	1.0 (.7–1.4)	1.0 (.8–1.4)	1.0 (.7–1.4)	1.1 (.9–1.3)
Not at All	1.4 (1.1–1.7)*	.9 (.7–1.2)	.9 (.6–1.3)	1.2 (.8–1.7)	1.1 (.7–1.8)	1.2 (.9–1.5)

*significance at *p* < .05

**Table 7:** Logistic regression models predicting health status and use of preventive measures by high school diploma status and health information source (referent category “A Lot”) for total sample, 2012 PIAAC data (N = 4885), Prostate, Dentist, Women and Men.

	Prostate	Dentist	Women**	Men***
	OR (95%CI)	OR (95%CI)	OR (95%CI)	OR (95%CI)
HIGH SCHOOL DIPLOMA	1.6 (.7–3.5)	1.3 (1.1–1.7)*	.9 (.6–1.2)	.9 (.4–2.0)
BOOKS				
Some	1.5 (.6–3.4)	.9 (.8–1.2)	1.2 (.9–1.6)	1.0 (.5–2.0)
A Little	1.3 (.6–2.9)	1.0 (.8–1.3)	1.3 (.9–1.7)	1.0 (.5–2.4)
Not at All	.8 (.4–2.0)	.9 (.7–1.2)	1.0 (.7–1.5)	1.3 .6–3.0)
HEALTH PROFESSIONALS				
Some	.8 (.5–1.1)	.9 (.8–1.0)	1.4 (1.1–1.9)*	1.6 (1.1–2.2)
A Little	.3 (.2-.6)*	.6 (.5-.8)*	2.1 (1.5–2.9)*	2.8 (1.6–4.9)
Not at All	.1 (.1-.3)*	.4 (.3-.6)*	5.4 (2.9–10.0)*	10.3 (4.6–23.0)
INTERNET				
Some	.8 (.5–1.3)	1.1 (.9–1.3)	.9 (.7–1.2)	1.2 (.8–1.6)
A Little	.9 (.4–1.8)	.8 (.7–1.0)	.8 (.6–1.2)	1.2 (.7–1.9)
Not at All	.9 (.5–1.5)	.6 (.5-.7)*	1.0 (.7–1.3)	1.1 (.6–1.8)
MAGAZINES				
Some	.8 (.3–2.0)	1.0 (.8–1.4)	.9 (.6–1.4)	.9 (.4–2.0)
A Little	1.0 (.4–2.7)	1.0 (.7–1.3)	1.0 (.6–1.5)	.9 (.4–2.0)
Not at All	1.0 (.4–2.4)	.8 (.6–1.1)	1.1 (.7–1.8)	1.1 (.5–2.4)
NEWSPAPER				
Some	.6 (.3–1.3)	1.1 (.8–1.4)	1.2 (.8–1.8)	1.7 (.8–3.3)
A Little	.6 (.3–1.3)	.9 (.7–1.3)	1.3 (.9–1.9)	1.6 (.8–3.2)
Not at All	.7 (.4–1.5)	.8 (.6–1.1)	1.4 (.9–2.2)	1.4 (.7–2.9)
RADIO				
Some	.9 (.5–1.8)	1.3 (.9–1.8)	.7 (.4–1.2)	1.0 (.5–2.1)
A Little	.8 (.4–1.8)	1.4 (1.1–1.9)*	.7 (.4–1.2)	1.0 (.5–2.4)
Not at All	.9 (.4–1.8)	1.6 (1.2–2.2)*	.7 (.4–1.1)	1.1 (.5–2.5)
TELEVISION				
Some	.9 (.6–1.5)	1.1 (.9–1.3)	1.0 (.7–1.3)	1.2 (.8–1.7)
A Little	1.4 (.8–2.4)	1.1 (.9–1.3)	1.1 (.8–1.5)	.9 (.6–1.4)
Not at All	1.4 (.7–2.9)	1.5 (1.2–1.9)*	1.1 (.7–1.6)	.8 (.4–1.7)

*significance at *p* < .05

**Women is a composite variable of the highly correlated variables mammogram, colonoscopy and osteoporosis screening

***Men is a composite variable of the highly correlated variables prostate screening and colonoscopy

Having a high school diploma was associated with increased odds of reporting excellent/very good health (1.34), getting a pap smear (1.57), and seeking dental care (1.33).

Those who did not use magazines a lot as a source of health information were less likely to get a pap smear (used some 38%, used a little 38%, did not use at all 54%). People who reported not using the Internet a lot were less likely to report excellent/very good health (used a little 26%, did not use at all 69%). They were also less likely to get a pap smear (did not use at all 27%) or get their vision checked (used some 17%, used a little 25%, did not use at all 29%). Those who did not use the Internet a lot were 42% less likely to have a dental visit.

Respondents who did not use the radio as a source of health information were more likely to get a flu shot than those who used the radio a lot (1.37 times). They were also more likely to get a mammogram if they listened to the radio some or not at all (1.75 and 1.83, respectively). Those who did not use television as a source of health information a lot were more likely to report excellent/very good health (used some 1.26, used a little 1.50, did not use at all 1.36) and were also more likely to have a dentist visit (a little 1.29, not at all 1.54) compared to those who used television a lot as a health source.

**Fig 1:**
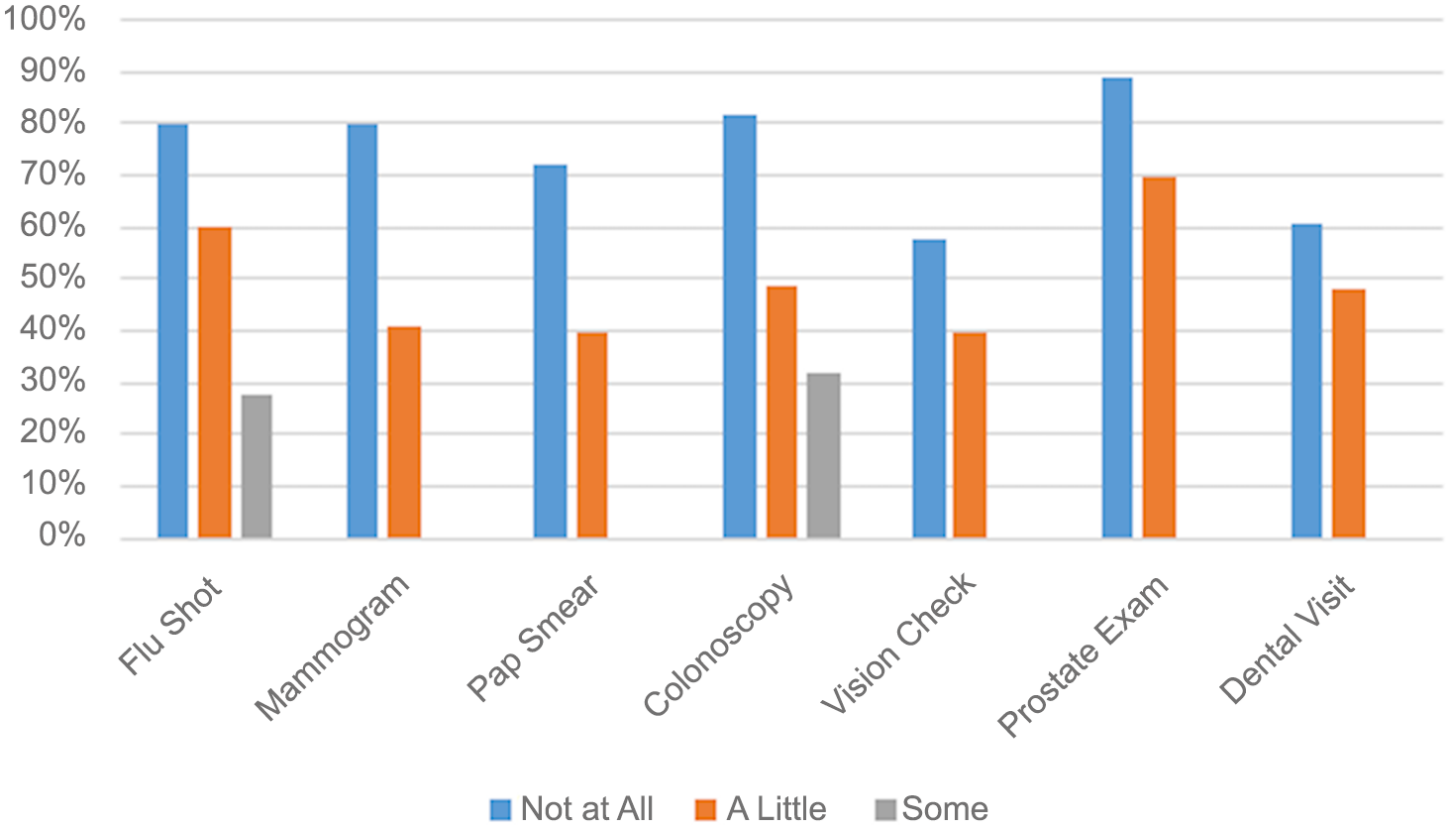
Reduced likelihood of using preventive measures for those who seek information from health professionals some, a little, or not at all, 2012 PIAAC data (N = 4885).

Respondents who sought information from health professionals a little or some were more likely to report excellent/very good health compared to those who sought health information from health professionals a lot (a little 1.36, some 1.29). Those who did not seek health information from health professionals or who only used this source a little or some were less likely to use preventive measures across all variables, significant at *p* < .05 (Fig 1).

These findings remained substantively unchanged with additional control for demographic covariates (results not shown).

We then assessed whether there were statistical interactions between having a high school diploma and the health information sources in relation to health status and preventive measures.

Interactions of HSD with seeking health information from health professionals (*p* = .03) and the Internet (*p* = .02) were found in relation to reporting excellent/very good health. Stratified models showed the association between Internet information use and excellent/very good health was similar for those with (OR = 0.44, 95% CI 0.35–0.57) and without (OR = 0.40, 95% CI 0.21–0.76) a HSD. Receiving information from health professionals and excellent/very good health status was positively associated for those with a HSD (OR 1.35, 95% CI 1.10–1.67) but non-significant for those with no HSD. Using magazines for health information for those with excellent/very good health was negatively associated (OR = 0.71, 95% CI 0.52–0.97) for those with a HSD but non-significant for those with no HSD.

An interaction of HSD by Internet health information use (*p* = .02) was associated with flu shots; however, the stratified models showed that Internet use was not significantly associated with getting flu shots for those with, nor without, a high school diploma. Using newspapers for health information Not at All or A Little for those who got a flu shot was negatively associated for those with a HSD only (OR = 0.66, 95% CI 0.44–0.99 and OR = 0.68 95% CI 0.47–0.98, respectively).

Significant interactions of HSD with seeking health information from health professionals (*p* = .01), the Internet (*p* = .01), and the radio (*p* = .02) were found in relation to getting pap smears, and stratified models showed that those who sought information from health professionals a lot had lower odds of getting pap smears among those without a high school diploma (OR = 0.06, 95% CI 0.01–0.27) compared to those with a high school diploma (OR = 0.42, 95% CI 0.26–0.68). Internet use was not associated with getting pap smears for those with and without high school diplomas. Those without a high school diploma who sought health information from the radio a lot were less likely to get a pap test (OR = 0.17, 95% CI 0.05–0.65), but no association was shown among those with a high school diploma.

An interaction of HSD with Internet use (*p* = .03) was found in relation to vision screenings, and stratified models showed that people without a high school diploma who do not use the Internet at all were significantly less likely to report having vision screenings (OR = 0.58, 95% CI 0.34–0.98), as were respondents with a high school diploma who use the Internet some (OR = 0.66, 95% CI 0.50–0.86). These results are compared to respondents who use the Internet A Lot.

There were several interactions for which the stratified models were inestimable. We therefore report no findings for these stratified models. They are: (1) an interaction of HSD with seeking health information from newspapers (*p* = .02) with colonoscopies, (2) interactions of having a high school diploma with health professionals (*p* = .003), magazine use (*p* = .02), and newspaper use (*p* = .01) for osteoporosis screenings, (3) interaction of HSD status with Internet use (*p* = .05) with the composite measure of preventive measures among women, and (4) an interaction of HSD status with reading books in relation to seeking dental care (*p* = .04).

## Discussion and Conclusions

### Discussion

Our study explored health information seeking behavior (HISB) and its relationship to health status and use of preventive measures, for those with and without a high school diploma while controlling for demographic factors. Our general findings indicate that while there is a difference in HISB between those with and those without a high school diploma, use of the Internet is a significant and important moderating factor. Internet use was related to better health status regardless of educational status; further, for those *without* a high school diploma, the health benefit of using the Internet as an information source was even greater than the benefit for those with a high school diploma. This suggests that for people both with and without a high school diploma, there may be positive health benefits to developing health-related digital literacy skills.

We first looked at the difference between uses of health information source by high school diploma status in Research Question 1. In all cases, there was a significant difference in the health information source category “A Lot of Use”. Those with a high school diploma were much more likely to use text-based sources while those without were much more likely to seek health information from oral sources (e.g., television and radio). This relationship held true when controlling for demographic factors of age, gender, race, immigrant status, and having medical insurance.

According to the PIAAC data, people with a high school diploma had a directly assessed mean reading literacy score of 276.0 versus 230.25 for those without a high school diploma (the US average is 270.0)[10]. Different reading skills are associated with each of these scores: the higher score indicates skills which are more complicated, include lengthy or dense texts, and the user can identify, interpret, evaluate and make appropriate references from one or more pieces of text while those with lower scores are more likely to only be able to access and identify information and make low-level inferences [10]. Written health information is often complex and dense, and is written in scientific jargon even when presented in an easier to read format [7,63, 75]. Those who have weak skills in navigating complex written text and in applying multi-step processes to understand, evaluate, and apply what is read may have difficulty accessing and using printed health materials [10].

We also found that people use multiple sources of health information. Our findings varied across health information sources, and between high school diploma status, although those with a high school diploma tended to use more information sources than those without a high school diploma. People may understand different aspects of health information differently, depending on whether it is media-related, people-related, actively sought, or passively sought [9, 76–78]. People also seek different types and amounts of information depending on their specific contexts and needs [64]. Health needs change over the course of a lifetime and it may be valuable to consider HISB as a continuum of information seeking rather than a discretely occurring behavior.

With regard to our second research question, we confirmed previous findings that having a high school diploma is related to having good health status [11–22,24,30,53,60] and that using the Internet for health information is related to having good health status [10,79–82]. However, unlike prior research, we were able to evaluate the interactive nature of Internet use for seeking health information, health status, use of preventive measures and high school diploma status to ascertain the particular cases where using the Internet has its most significant impact. A key finding is the strong association between digital literacy and health status regardless of educational status.

The Internet is the fasting growing source of health information [83,84] and is widely used by those providing health services such as insurance companies, pharmaceutical companies, hospitals, physicians, wellness providers, employers, and others. The high literacy demands of health-related websites create problems in understanding and applying information, even for those who have a high school diploma [68]. Adults of all education levels may have difficulty searching for health information on the Internet due to inability to generate effective search terms, an aversion to using links on web pages, access to computers, and difficulty understanding how to use the information obtained [84]. In addition, health information that people acquire from the Internet is often neither accurate nor complete. Despite these barriers, and despite educational attainment, those who do use the Internet a lot report excellent/very good health status more than those who use any other source.

People who do not use the Internet may face a critical gap in accessing health information as more health professionals and consumer organizations and agencies rely on its use. Digital literacy includes both use of physical technology and having the literacy skills to search and access information including medical communication such as medical forms, insurance forms, Internet search terms, and screening guidelines. The application of digital technology in the health domain can widen the digital divide between those who may not have access due to socioeconomic discrepancies [84–86]. Patterns of knowledge consumption and use may also be different for those with and without a high school diploma. For example, explicit and implicit knowledge and learning occur in formalized learning settings such as high schools, and those who have not completed high school may not have the benefit of skills development in this area.

We also were interested in knowing if health information seeking behavior was associated with use of preventive measures. While use of the Internet was most significant in having excellent/very good health status, the strongest use of preventive measures occurred when people used health professionals as their information source. Another finding suggest that women who used magazines as a health information source were much more likely to get Pap Tests. Women’s health issues have been prevalent in mass media, which continues to provide positive influences on health choices; 7 of the top 10 magazines in 2011 circulation were related to women and families [87].

### Implications

The implications from our study are threefold—access, education, and ease of use. Our results suggest the importance of development of digital literacy skills for seeking health-related information for all people, regardless of educational status. The traditional digital divide still exists and those with lower usage of the Internet and computers lag far behind; heavily linked with the obstinate social impediments of race, poverty, and education, reducing the gap should be a top issue of particular urgency to both the public and private sectors [83,85,88,89].

Over 1.8 million adults attend Adult Literacy classes in order to gain literacy skills [89]. Along with reading and numeracy, digital literacy may be a remediable skill for adults in adult education programs; further, enhancing skill level and self-efficacy in digital literacy could significantly change how adults with low literacy levels seek and use health information [85,89]. There is little curricular guidance, professional development, and overall funding provided to Adult Literacy providers that allows them to include much more than basic literacy and GED skills training. The implications of low health literacy have come to the attention of the healthcare community over the last 20 years. While increasing attention has been paid to the readability levels of printed materials [8], the simultaneous increase of health information on the Internet confounds this advance. Health promotion and education interventions and materials should be developed at appropriate literacy levels, so that all patients and consumers can access the information digitally.

### Limitations

Our findings should be considered in light of some limitations. The PIAAC data is cross-sectional, therefore we are only able to determine if there is association between variables, not causality. Health status and use of preventive measures were self-reported variables, and we had no relevant clinical data to support the respondents’ claims. Analyzing ordinal data required that we meet odds ratio assumptions; these assumptions were violated in several analyses, and we had to collapse variables into dichotomous categories, particularly with regard to use of preventive measures. Additionally, not every preventive measure is performed each year, e.g., colonoscopies are generally on a 5-year schedule, so a person may have had a colonoscopy 2 years prior to the survey and they would not report it during the last 12 months even though they were compliant with a preventive measure. We also faced sample size issues when evaluating health status: it was necessary to collapse health status data into two groupings—excellent/very good and good/fair/poor—in order to complete our analysis. In addition, we did not stratify across age groups, which could have some impact on use of health information sources such as the Internet since research shows that younger adults are heavier Internet users [90,91]. Although we controlled for race, we did not stratify across racial categories to determine its impact on HISB, health status, and use of preventive measures. We also did not examine digital access or income levels for the respondents, which could create differentiated usage patterns of health information sources. However, despite these limitations, our study is the first to provide valuable information in understanding the relationships between educational attainment, health information seeking, health status and use of preventive measures through analysis of the U.S. PIAAC data.

## Supporting Information

S1 File (DOCX)
