# Supplementary material for: Examining Associations between Health Information Seeking Behavior and Adult Education Status in the U.S.: An Analysis of the 2012 PIAAC Data
Source: PLoS One. 2016 Feb 16;11(2):e0148751. doi: 10.1371/journal.pone.0148751 (PMC4755661; doi:10.1371/journal.pone.0148751)
Supplement: S1 File — (DOCX) [file pone.0148751.s001.docx]

**Appendix**

| **Table A. Interactions Model 1 HISB Sources and Magazine Usage by HSD**. 2012 PIAAC Data (N=4885) | | | | | |
| --- | --- | --- | --- | --- | --- |
|  |  | No HSD Diploma | | HSD Diploma | |
|  |  | OR | 95% CI | OR | 95% CI |
| Newspaper | Not At All (ref) | |  |  |  |
|  | Little | 3.59 | (2.01-6.41) | 2.34 | (1.91-2.88) |
|  | Some | 7.82 | (4.25-14.38) | 6.73 | (5.56-8.16) |
|  | A Lot | 43.78 | (10.92-175.59) | 19.12 | (12.86-28.44) |
| Magazines | Not At All (ref) | |  |  |  |
|  | Little |  |  |  |  |
|  | Some |  |  |  |  |
|  | A Lot |  |  |  |  |
| Internet | Not At All (ref) | |  |  |  |
|  | Little | 1.33 | (0.56-3.13) | 1.23 | (0.87-1.74) |
|  | Some | 2.04 | (1.07-3.88) | 1.81 | (1.37-2.41) |
|  | A Lot | 2.14 | (1.21-3.77) | 2.08 | (1.51-2.88) |
| Radio | Not At All (ref) | |  |  |  |
|  | Little | 1.01 | (0.63-1.62) | 1.26 | (1.06-1.51) |
|  | Some | 1.45 | (0.80-2.64) | 1.19 | (0.97-1.46) |
|  | A Lot | 1.56 | (0.87-2.80) | 1.33 | (1.03-1.72) |
| TV | Not At All (ref) | |  |  |  |
|  | Little | 2.7 | (1.02-7.11) | 1.59 | (1.17-2.15) |
|  | Some | 2.78 | (1.06-7.33) | 2 | (1.52-2.63) |
|  | A Lot | 2.12 | (0.78-5.77) | 2.28 | (1.74-2.99) |
| Books | Not At All (ref) | |  |  |  |
|  | Little | 3.57 | (2.09-6.12) | 3 | (2.46-3.66) |
|  | Some | 4.75 | (2.35-9.60) | 4.97 | (4.07-6.06) |
|  | A Lot | 8.46 | (4.02-17.79) | 9.41 | (6.66-13.29) |
| Health Professionals | Not At All (ref) | |  |  |  |
|  | Little | 0.81 | (0.33-1.97) | 1.1 | (0.78-1.55) |
|  | Some | 0.93 | (0.41-2.12) | 0.95 | (0.69-1.30) |
|  | A Lot | 1.04 | (0.47-2.31) | 0.78 | (0.57-1.07) |
|  |  |  |  |  |  |

| **Table B. Interactions Model 2 HISB Sources and Internet Usage by HSD**. 2012 PIAAC data (N=4885) | | | | | |
| --- | --- | --- | --- | --- | --- |
|  |  | No HSD Diploma | | HSD Diploma | |
|  |  | OR | 95% CI | OR | 95% CI |
| Newspaper | Not At All (ref) | |  |  |  |
|  | Little | 1.29 | (0.77-2.16) | 0.84 | (0.73-0.98) |
|  | Some | 1.28 | (0.68-2.40) | 0.81 | (0.66-0.99) |
|  | A Lot | 0.85 | (0.30-2.41) | 0.94 | (0.62-1.41) |
| Magazines | Not At All (ref) | |  |  |  |
|  | Little | 1.53 | (0.95-2.47) | 1.65 | (1.29-2.12) |
|  | Some | 2.09 | (1.14-3.81) | 1.85 | (1.41-2.44) |
|  | A Lot | 2.3 | (0.94-5.66) | 2.47 | (1.83-3.33) |
| Internet | Not At All (ref) | |  |  |  |
|  | Little |  |  |  |  |
|  | Some |  |  |  |  |
|  | A Lot |  |  |  |  |
| Radio | Not At All (ref) | |  |  |  |
|  | Little | 1.42 | (0.85-2.36) | 1.24 | (1.04-1.48) |
|  | Some | 1.36 | (0.88-2.11) | 1.41 | (1.15-1.72) |
|  | A Lot | 2.51 | (0.83-7.55) | 1.51 | (1.06-2.15) |
| TV | Not At All (ref) | |  |  |  |
|  | Little | 1.02 | (0.47-2.20) | 1.14 | (0.91-1.44) |
|  | Some | 1.16 | (0.56-2.42) | 1.15 | (0.91-1.45) |
|  | A Lot | 1.51 | (0.70-3.25) | 1.16 | (0.88-1.53) |
| Books | Not At All (ref) | |  |  |  |
|  | Little | 1.83 | (1.12-2.99) | 1.92 | (1.50-2.45) |
|  | Some | 3.84 | (2.16-6.83) | 1.96 | (1.58-2.44) |
|  | A Lot | 2.53 | (1.19-5.35) | 2.4 | (1.87-3.08) |
| Health Professionals | Not At All (ref) | |  |  |  |
|  | Little | 0.86 | (0.34-2.16) | 1.71 | (1.14-2.58) |
|  | Some | 1.34 | (0.60-3.01) | 2.06 | (1.31-3.22) |
|  | A Lot | 1.16 | (0.49-2.74) | 1.95 | (1.25-3.06) |
|  |  |  |  |  |  |

| **Table C. Interactions Model 4 HISB Sources and Television Usage by HSD**. 2012 PIAAC data (N=4885) | | | | | | |
| --- | --- | --- | --- | --- | --- | --- |
|  |  | No HSD Diploma | | HSD Diploma | |  |
|  |  | OR | 95% CI | OR | 95% CI |  |
| Newspaper | Not At All (ref) | |  |  |  |  |
|  | Little | 1.06 | (0.61-1.84) | 1.25 | (1.04-1.50) |  |
|  | Some | 1.08 | (0.66-1.75) | 1.48 | (1.17-1.87) |  |
|  | A Lot | 3 | (1.00-8.99) | 2.3 | (1.63-3.23) |  |
| Magazines | Not At All (ref) | |  |  |  |  |
|  | Little | 1.51 | (0.97-2.35) | 1.37 | (1.08-1.75) |  |
|  | Some | 0.92 | (0.59-1.44) | 1.71 | (1.32-2.22) |  |
|  | A Lot | 1.91 | (0.98-3.71) | 2.12 | (1.60-2.82) |  |
| Internet | Not At All (ref) | |  |  |  |  |
|  | Little | 0.76 | (0.38-1.51) | 0.68 | (0.52-0.90) |  |
|  | Some | 0.8 | (0.42-1.54) | 0.64 | (0.48-0.86) |  |
|  | A Lot | 1.37 | (0.75-2.53) | 0.82 | (0.62-1.09) |  |
| Radio | Not At All (ref) | |  |  |  |  |
|  | Little | 1.61 | (1.06-2.46) | 1.59 | (1.34-1.90) |  |
|  | Some | 3.995 | (2.48-6.44) | 2.78 | (2.32-3.35) |  |
|  | A Lot | 7.28 | (4.23-12.53) | 6.26 | (4.28-9.15) |  |
| TV | Not At All (ref) | |  |  |  |  |
|  | Little |  |  |  |  |  |
|  | Some |  |  |  |  |  |
|  | A Lot |  |  |  |  |  |
| Books | Not At All (ref) | |  |  |  |  |
|  | Little | 0.79 | (0.49-1.27) | 1.13 | (0.89-1.44) |  |
|  | Some | 1.1 | (0.63-1.90) | 1.08 | (0.84-1.40) |  |
|  | A Lot | 0.95 | (0.51-1.76) | 0.9 | (0.64-1.25) |  |
| Health Professionals | Not At All (ref) | |  |  |  |  |
|  | Little | 1.17 | (0.63-2.18) | 1.21 | (0.81-1.79) |  |
|  | Some | 1.21 | (0.56-2.60) | 1.23 | (0.82-1.83) |  |
|  | A Lot | 1.26 | (0.61-2.63) | 1.23 | (0.84-1.81) |  |

| **Table D. Interactions Model 5 HISB Sources and Book Usage by HSD**. 2012 PIAAC data (N=4885) | | | | | |  |
| --- | --- | --- | --- | --- | --- | --- |
|  |  | No HSD Diploma | | HSD Diploma | | |
|  |  | OR | 95% CI | OR | 95% CI | |
| Newspaper | Not At All (ref) | |  |  |  | |
|  | Little | 1.01 | (0.57-1.77) | 1.21 | (1.02-1.43) | |
|  | Some | 1.45 | (0.70-3.00) | 1.33 | (1.07-1.66) | |
|  | A Lot | 1.51 | (0.68-3.33) | 1.82 | (1.37-2.42) | |
| Magazines | Not At All (ref) | |  |  |  | |
|  | Little | 4.21 | (2.30-7.71) | 2.83 | (2.23-3.60) | |
|  | Some | 5.79 | (3.03-11.04) | 4.6 | (3.59-5.89) | |
|  | A Lot | 11.74 | (4.19-32.84) | 14.03 | (9.41-20.91) | |
| Internet | Not At All (ref) | |  |  |  | |
|  | Little | 2.25 | (1.19-4.28) | 1.53 | (1.16-2.01) | |
|  | Some | 3.47 | (1.35-4.51) | 1.91 | (1.49-2.44) | |
|  | A Lot | 3.4 | (1.80-6.42) | 2.17 | (1.69-2.79) | |
| Radio | Not At All (ref) | |  |  |  | |
|  | Little | 1.05 | (0.62-1.81) | 1 | (0.86-1.17) | |
|  | Some | 1.57 | (0.87-2.83) | 1.29 | (1.06-1.57) | |
|  | A Lot | 1.31 | (0.63-2.70) | 1.29 | (1.01-1.65) | |
| TV | Not At All (ref) | |  |  |  | |
|  | Little | 0.93 | (0.43-2.12) | 0.96 | (0.74-1.23) | |
|  | Some | 1.04 | (0.46-2.34) | 0.96 | (0.74-1.23) | |
|  | A Lot | 0.89 | (0.63-1.25) | 0.88 | (0.66-1.19) | |
| Books | Not At All (ref) | |  |  |  | |
|  | Little |  |  |  |  | |
|  | Some |  |  |  |  | |
|  | A Lot |  |  |  |  | |
| Health Professionals | Not At All (ref) | |  |  |  | |
|  | Little | 2.61 | (1.24-5.48) | 1.8 | (1.29-2.50) | |
|  | Some | 2.64 | (1.37-5.09) | 2.34 | (1.72-3.32) | |
|  | A Lot | 3.35 | (1.64-6.81) | 4.87 | (3.55-6.68) | |
|  |  |  |  |  |  | |
